# Machine Learning-Based Prediction Models for Depression Symptoms Among Chinese Healthcare Workers During the Early COVID-19 Outbreak in 2020: A Cross-Sectional Study

**DOI:** 10.3389/fpsyt.2022.876995

**Published:** 2022-04-29

**Authors:** Zhaohe Zhou, Dan Luo, Bing Xiang Yang, Zhongchun Liu

**Affiliations:** ^1^School of Basic Medical Sciences, Chengdu University, Chengdu, China; ^2^School of Nursing, Wuhan University, Wuhan, China; ^3^Population and Health Research Center, Wuhan University, Wuhan, China; ^4^Department of Psychiatry, Renmin Hospital of Wuhan University, Wuhan, China

**Keywords:** depression, machine learning, COVID-19, health personnel, predictive value of tests

## Abstract

**Background:**

The 2019 novel coronavirus (COVID-19)-related depression symptoms of healthcare workers have received worldwide recognition. Although many studies identified risk exposures associated with depression symptoms among healthcare workers, few have focused on a predictive model using machine learning methods. As a society, governments, and organizations are concerned about the need for immediate interventions and alert systems for healthcare workers who are mentally at-risk. This study aims to develop and validate machine learning-based models for predicting depression symptoms using survey data collected during the COVID-19 outbreak in China.

**Method:**

Surveys were conducted of 2,574 healthcare workers in hospitals designated to care for COVID-19 patients between 20 January and 11 February 2020. The patient health questionnaire (PHQ)-9 was used to measure the depression symptoms and quantify the severity, a score of ≥5 on the PHQ-9 represented depression symptoms positive, respectively. Four machine learning approaches were trained (75% of data) and tested (25% of data). Cross-validation with 100 repetitions was applied to the training dataset for hyperparameter tuning. Finally, all models were compared to evaluate their predictive performances and screening utility: decision tree, logistics regression with least absolute shrinkage and selection operator (LASSO), random forest, and gradient-boosting tree.

**Results:**

Important risk predictors identified and ranked by the machine learning models were highly consistent: self-perceived health status factors always occupied the top five most important predictors, followed by worried about infection, working on the frontline, a very high level of uncertainty, having received any form of psychological support material and having COVID-19-like symptoms. The area under the curve [95% CI] of machine learning models were as follows: LASSO model, 0.824 [0.792–0.856]; random forest, 0.828 [0.797–0.859]; gradient-boosting tree, 0.829 [0.798–0.861]; and decision tree, 0.785 [0.752–0.819]. The calibration plot indicated that the LASSO model, random forest, and gradient-boosting tree fit the data well. Decision curve analysis showed that all models obtained net benefits for predicting depression symptoms.

**Conclusions:**

This study shows that machine learning prediction models are suitable for making predictions about mentally at-risk healthcare workers predictions in a public health emergency setting. The application of multidimensional machine learning models could support hospitals' and healthcare workers' decision-making on possible psychological interventions and proper mental health management.

## Introduction

Since the first confirmed 2019 novel coronavirus (COVID-19) infection case, the COVID-19 pandemic has brought tremendous challenges to the global healthcare system (1). Facing this global pandemic, healthcare workers bear the brunt of this aggravating healthcare burden. Healthcare workers, especially doctors and nurses who directly care for COVID-19 patients, are at great risk of developing mental health illnesses (2). COVID-19-related mental health problems for healthcare workers have received high attention in academia (3–5). Recent meta-studies found depression is the most common mental health outcome among healthcare workers due to the impact of COVID-19, with a prevalence rate of 20–30% worldwide (6–9).

The theory of crisis used as a theoretical framework guided this study. According to James and Gilliland, “crisis” is a broad and subjective term used to describe a situation that affects an individual excruciatingly due to various life, environmental, and psychological stressors. In addition, substantial evidence from the previous studies of epidemics on the impact of psychological health has shown psychosocial consequences in the affected individuals and the general population. In this study, COVID-19 is considered a crisis that leads to intense psychosocial issues and comprises mental health marking a secondary health concern worldwide. The research findings helped us to cultivate risk factors associated with depression symptoms among healthcare workers, namely, disease-related exposures (10), worried about infection (10), working on the frontline (11, 12), gender differences (11, 13, 14), type of hospital (11), technical title (11), location (14), lacking social support (15), and uncertainty toward the pandemic (16).

While most studies focused on understanding the exposure-outcome association of depression symptoms, research on identifying signs that predict depression symptoms were limited. The WHO recommended “psychological first aid” (17), which promotes immediate help and support to field workers who are experiencing mental disorders due to a recent crisis. Furthermore, machine learning, an innovative approach, has extensive applications in prediction to identify patients at high risk, their death rate, and other abnormalities during the pandemic of COVID-19 (18, 19). In a previous study, machine learning functioned as a valuable technique to suppress interferences out of unlabeled input datasets, which can be applied to analyze the unlabeled data as an input resource for COVID-19 (20). Machine learning techniques provide accurate and useful features rather than a traditional explicitly calculation-based method (21). It is also beneficial to predict the risk in healthcare during this COVID-19 crisis and analyze the risk factors as per age, social habits, location, and climate (22). However, in mental health prediction, the application of machine learning is still in preliminary status. If machine learning models can predict depression symptoms in a timely manner and are available in a clinical setting following a short survey, they can serve as a self-screening mechanism to alert healthcare management about employees who are at risk of depression. The unique variable importance feature of machine learning models can be used to help develop immediate interventions for healthcare workers in preparation for the next public health emergency. To the best of our knowledge, machine learning models were rarely used to predict COVID-19-related mental health outcomes of healthcare workers.

To address this gap, the goal of this cross-sectional study is to develop machine learning models using quantified questionnaire data that can efficiently predict depression symptoms in healthcare workers using the following machine learning techniques: decision tree, logistic regression with least absolute shrinkage and selection operator (LASSO), random forest, and gradient-boosting trees. In addition, the models can help determine psychological and behavioral factors that place healthcare workers at-risk for alterations in mental health and to access the needs of healthcare workers during a public health emergency. The predictive performance and screening utility among these models are also compared and assessed.

## Methods

### Participants and Data Collection

This national survey was conducted in Chinese using the WeChat-based online survey platform “Wenjuanxing” between 20 January and 11 February 2020. The survey was distributed in WeChat, a widely used social communication application. Staff from the COVID-19 designated hospitals were contacted by the researcher and asked to invite healthcare workers in their facility to complete this online survey. The eligibility criteria of participants were: licensed healthcare personnel and working in a hospital designated to care for COVID-19 patients. This survey was accessed by a large population of healthcare workers. All participants were asked to complete an online informed consent before completing the survey. A total of 2,574 healthcare workers completed the survey. Ethical approval for this study was received from the Institutional Review Board at Renmin Hospital of Wuhan University (No. WDRY2020-K004).

### Variables and Measurements

The questionnaire includes sociodemographic characteristics and other items regarding mental health outcomes, COVID-19 exposures, use of psychological services, information channels, perception of the pandemic, and self-perceived health status total of 8 segments and 23 potential exposures.

### Sociodemographic Characteristics

Includes information on frontline work (Yes/No), gender, education level, marital status, geographic location, living arrangements, and age groups.

### COVID-19 Exposure

Questions include: Have you or your family members been diagnosed with COVID-19? Have you had a COVID-19-like symptom (fever, dry cough, fatigue, etc.)? Do you worry about being infected? Have your colleagues been diagnosed with COVID-19? Have your friends been diagnosed with COVID-19? Have people in your neighborhood been diagnosed with COVID-19?

### Psychological Services

Questions are related to access to psychological help: Have you received any form of psychotherapy, both one-to-one and group-based? Have you received any form of psychological support material, both paper-based and media-based? Have you received any other psychological help?

### Media Usage

Questions focus on the type of media used and the amount of time spent obtaining information on COVID-19: Do you get COVID-19 information talking/chatting with others? Do you get COVID-19 information from television? Do you get COVID-19 information through new media like WeChat, TikTok, Weibo, etc.? On average, how long did you spend each day seeking COVID-19 information?

### Perception of Pandemic

The question focused on personal views on resolving the pandemic: What is your belief about whether the pandemic can be controlled: very strong, strong, normal, and none.

### Self-Perceived Health Status

The final segment asks healthcare workers to self-report their health status: describe your current health status. What is your current health status now compared to your health status before the outbreak?

### Mental Health Outcomes

Depression was evaluated using the Chinese version of the patient health questionnaire (PHQ-9) (23), which has nine items measuring self-assessed depressive symptoms experienced during the previous 2 weeks. It uses a 4-point Likert-type scale (0 = never, 1 = sometimes, 2 = more than once a week, and 3 = almost every day). The total score ranges from 0 to 27, and higher scores indicate more depressive symptoms. Scores of 10 and 15 represent cutpoints for moderate and moderately severe depression, respectively. The Chinese version of the PHQ-9 has shown good psychometric properties with reported Cronbach's α of 0.86 (24).

### Statistical Analysis

The primary outcome of the study is a depression event, defined as a score on the PHQ-9 ≥ 5. Descriptive statistics include the frequency and percentage of depression symptoms under each potential predictor. This aims to provide characteristics for the entire population.

Based on the predictive performances of previous depression-related mental health or COVID-19-related predictions (25–31), four machine learning techniques were developed: decision tree (28, 30), logistics regression with least LASSO (25, 26), random forest (28–30), and gradient-boosting tree (27, 28, 31). Although details of these machine learning techniques are well documented, brief descriptions for each model and hyperparameter are carried out below.

### Logistic Regression With LASSO

Logistic regression with LASSO chooses relative important predictors out of all possible predictors by not only minimizing the residual sum of square (RSS) of the coefficient, just like the ordinary least square regression method, but adding a penalty to the RSS equal to the sum of the absolute value as well (it shrinks some coefficient estimates toward zero) (32–34). The hyperparameter “lambda” controls the penalty to the residual sum of a square and was optimized during the cross-validation process. The hyperparameter “alpha” is for the elastic net mixing parameter, hence we set alpha equal to 1 in LASSO regression.

### Decision Tree

A decision tree recursively splits a parent node using a finite number of potential predictors stopped by reaching the minimum cost complexity (this process is also called pruning), which results in outcome classification (35). The cost complexity is measured by the number of leaves in the tree (size of the tree) and the error rate of the tree (misclassification rate). The hyperparameter “complexity parameter” refers to the amount by which splitting a node improved the relative error. In other words, the decision tree tries to have the smallest tree with the smallest cross-validation error and its complexity parameter is the trade-off threshold between the size of the tree and the misclassification rate to help prevent overfitting.

### Random Forest

Random forest is an ensemble learning method that constructs many independent decision trees without pruning and produces a single estimate by combining every tree's predictions (36). The permutation method in the random forest is used to access the importance of predictors by comparing prediction accuracy differences between the results from permuting variables in out-of-bag samples and the result without permutation. Instead of doing an exhaustive search over all potential predictors, random forest randomly sampled “mtry” variables as candidate predictors when forming each split in a tree. The hyperparameter “mtry” was optimized during the cross-validation process. To make ensemble tree methods comparable to each other, the researchers set the other hyperparameter, the number of the tree built, to the fixed 1,000 trees.

### Gradient-Boosting Tree

In contrast, a gradient-boosting tree constructs one tree sequentially that aims to improve the shortcomings of the previous tree at each iteration. Importance is determined by the relative influence of each predictor: whether that predictor was selected to split on and how much the squared error improved (37–39). To prevent overfitting, the complexity of the tree at each iteration was controlled by three hyperparameters: the minimum number of observations in the terminal nodes, max tree depth, and shrinkage. To make ensemble tree methods comparable to each other, the researchers set the other hyperparameter, the number of the tree built, to the fixed 1,000 trees.

Except for reporting the beta coefficient of the LASSO method, the researchers scaled each tree-based variable importance unit by the maximum value of 100 to give a straightforward understanding of the sense of variable importance.

### Model Training

During the training, the data were randomly split into a 75% training dataset and a 25% test dataset. The training dataset was used to train and validate each of the four models. For each type of model, the hyperparameters were optimized using 100 repetitions of grid search and evaluating the results using three-fold cross-validation. Once the optimal hyperparameters were determined, each model has fitted again on the entire training dataset. The optimal hyperparameters are reported in Supplementary Table 1. All training was supervised, meaning the depression outcomes were provided during the training.

### Performance Measurement

The test dataset was used to test and compare each of the four models' performances from the perspective of prediction accuracy and screening utility. Receiver operating characteristic (ROC) was used as the main measure (40) of prediction accuracy alone with the nonparametric DeLong test to compare the area under the curve (AUC) among the models (41). To assess the model fit, calibrations were plotted to observe the consistency between model-produced probabilities and observed probabilities of depression events. Finally, screening utility was assessed by calculating net-benefit values (42, 43), the differences between the proportion of true positive counts (benefit) and weighted proportion of false-positive counts (harm) at each probability threshold of a depression event; the decision curves were plotted as well.

All computations were performed using R (version 3.5.0); R package “haven” was employed for importing data. The R package “caret” was used for tuning hyperparameters during the model training. The R package “rpart” was employed for performing a decision tree. The R package “glmnet” was used for performing LASSO. The R package “randomForest” was employed for performing random forest. The R package “gbm” was used for performing a gradient-boosting decision tree. The R package “pROC” was employed for performing ROC analysis. All tests were two-sided and considered statistically significant if the *p*-value was < 0.05.

## Results

### Participants Characteristics

**Figure 1:**
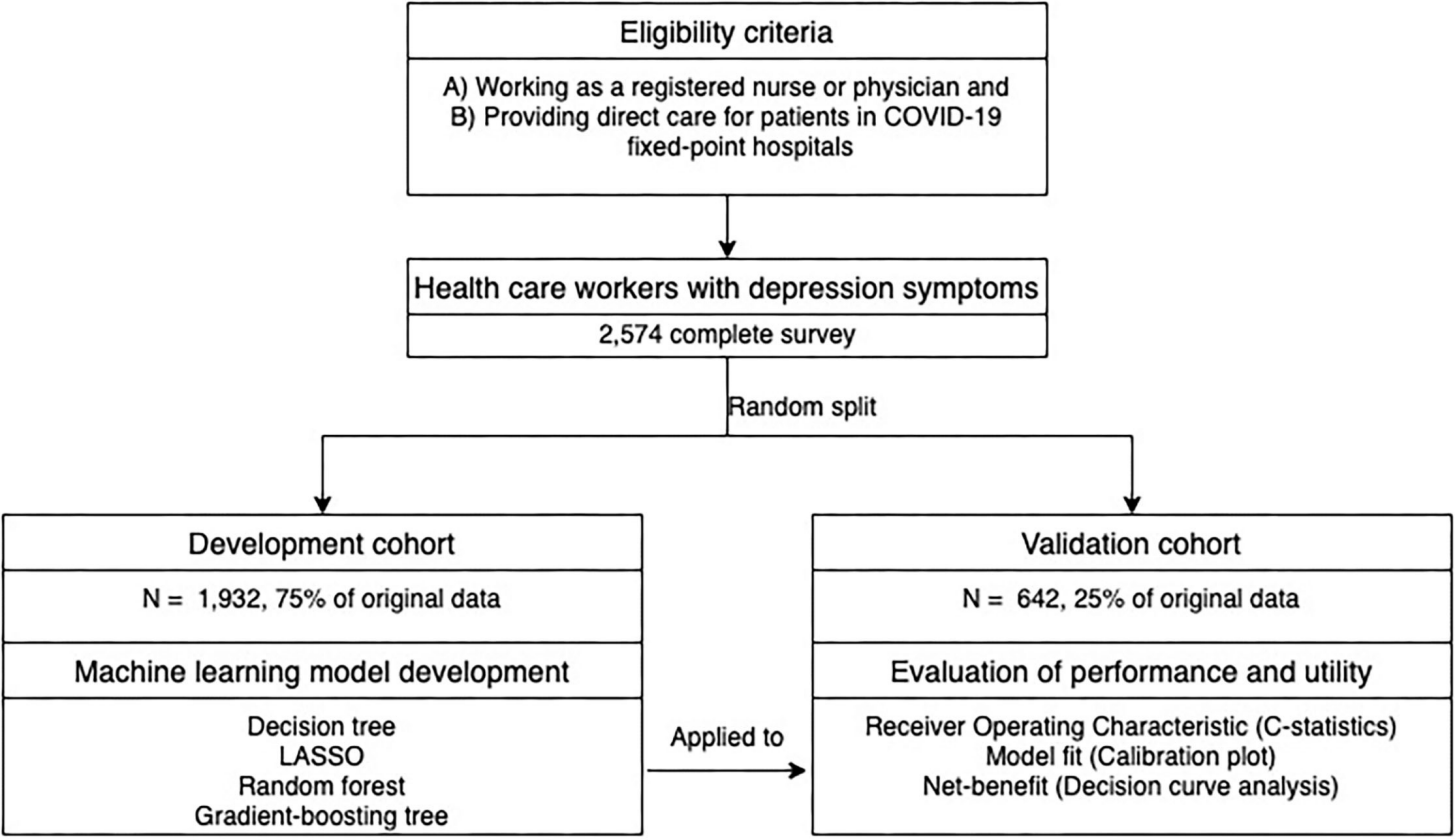
Study flowchart.

The study flowchart and participants' characteristics are summarized in Figure 1 and Table 1, respectively. In total, questionnaires from 2,574 healthcare workers were analyzed; this includes 1,187 participants (46.11%) with depression symptoms. The participants were randomly split into the training dataset (*N* = 1,932) and the test dataset (*N* = 642). The participants' characteristics were as follows: participants were predominantly female, holding an undergraduate degree or below, worried about infection, without COVID-19-like symptoms, getting information through new media, most of them did not receive any professional psychological therapy, health status getting worse, and without infection exposure (self and others); the majority were married, living with family, spending 1–2 h daily seeking COVID-19 information. The rest of the characteristics generally were evenly distributed within each question item.

**Table 1:** Demographic and variable characteristics.

**Variables**		**Training dataset**		**Test dataset**	
		**No depression symptoms** **(*n =* 1,041)**	**Have depression symptoms (*n =* 891)**	**No depression symptoms** **(*n =* 346)**	**Have depression symptoms** **(*n =* 296)**
**Gender**					
	Female	783 (75.2%)	746 (83.7%)	266 (76.9%)	241 (81.4%)
	Male	258 (24.8%)	145 (16.3%)	80 (23.1)	55 (18.6%)
**Frontline worker**					
	Yes	279 (26.8%)	406 (45.6%)	98(28.3%)	132(44.6%)
	No	762 (73.2%)	485 (54.4%)	248(71.7%)	164(55.4%)
**Married**					
	Yes	715 (68.7%)	548 (61.5%)	238 (68.8%)	197 (66.6%)
	No	326 (31.3%)	343 (38.5%)	108 (31.2%)	99 (33.4%)
**Education**					
Graduate degree or higher		177 (17.0%)	154 (17.3%)	66(19.1%)	51(17.2%)
Undergraduate degree or lower		864 (83.0%)	737 (82.7%)	280(80.9%)	245(82.8%)
**COVID-19-like symptom (fever, dry cough, fatigue etc.)**					
	Yes	126 (12.1%)	194 (21.8%)	37 (10.7%)	68 (23.0%)
	No	915 (87.9%)	697 (78.2%)	309 (89.3%)	228 (77.0%)
**Worry about infection**					
	Yes	740 (71.1%)	819 (91.9%)	228 (65.9%)	268 (90.5%)
	No	301 (28.9%)	72 (8.1%)	118 (34.1%)	28 (9.5%)
**Getting COVID-19 information via talking/chatting**					
	Yes	572 (54.9%)	562 (63.1%)	204 (59.0%)	195 (65.9%)
	No	469 (45.1%)	329 (36.9%)	142 (41.0%)	101 (34.1%)
**Getting COVID-19 information through new media (WeChat, TikTok, Weibo, etc.)**					
	Yes	997 (95.8%)	861 (96.6%)	329 (95.1%)	283 (95.6%)
	No	44 (4.2%)	30 (3.4%)	17 (4.9%)	13 (4.4%)
**Getting COVID-19 information from television**					
	Yes	574 (55.1%)	429 (48.1%)	202 (58.4%)	148 (50.0%)
	No	467 (44.9%)	462 (51.9%)	144 (41.6%)	148 (50.0%)
**Received any form of psychotherapy**					
	Yes	141 (13.5%)	125 (14.0%)	52 (15.0%)	48 (16.2%)
	No	900 (86.5%)	766 (86.0%)	294 (85.0%)	248 (83.8%)
**Received any form of psychological support material**					
	Yes	672 (64.6%)	451 (50.6%)	228 (65.9%)	149 (50.3%)
	No	369 (35.4%)	440 (49.4%)	118 (34.1%)	147 (49.7%)
**Received other psychological help**					
	Yes	42 (4.0%)	45 (5.1%)	23 (6.6%)	11 (3.7%)
	No	999 (96.0%)	846 (94.9%)	323 (93.4%)	285 (96.3%)
**Living arrangement**					
	Live with family	712 (68.4%)	484 (54.3%)	249 (72.0%)	170 (57.4%)
	Live alone	214 (20.6%)	221 (24.8%)	63 (18.2%)	72 (24.3%)
	Live with friends	108 (10.4%)	168 (18.9%)	33 (9.5%)	51 (17.2%)
	Live with others	7 (0.7%)	18 (2.0%)	1 (0.3%)	3 (1.0%)
**Location of residence**					
	Wuhan city	377 (36.2%)	459 (51.5%)	112 (32.4%)	154 (52.0%)
	Hubei province	297 (28.5%)	172 (19.3%)	103 (29.8%)	68 (23.0%)
	Other province	367 (35.3%)	260 (29.2%)	131 (37.9%)	74 (25.0%)
**Time spent seeking COVID-19 information**					
	<1 h	206 (19.8%)	144 (16.2%)	58 (16.8%)	34 (11.5%)
	1–2 h	473 (45.4%)	336 (37.7%)	166 (48.0%)	121 (40.9%)
	3–4 h	226 (21.7%)	218 (24.5%)	68 (19.7%)	83 (28.0%)
	Over 5 h	136 (13.1%)	193 (21.7%)	54 (15.6%)	58 (19.6%)
**Perception on pandemic control**					
	Very strong	117 (11.2%)	237 (26.6%)	39 (11.3%)	69 (23.3%)
	Strong	338 (32.5%)	339 (38.0%)	119 (34.4%)	127 (42.9%)
	Normal	538 (51.7%)	306 (34.3%)	178 (51.4%)	97 (32.8%)
	None	48 (4.6%)	9 (1.0%)	10 (2.9%)	3 (1.0%)
**Self-perceived health status compered to before COVID-19 outbreak**					
	Much worse	91 (8.7%)	312 (35.0%)	22 (6.4%)	110 (37.2%)
	Worse	867 (83.3%)	499 (56.0%)	288 (83.2%)	162 (54.7%)
	Unchanged	76 (7.3%)	33 (3.7%)	34 (9.8%)	7 (2.4%)
	Better	7 (0.7%)	47 (5.3%)	2 (0.6%)	17 (5.7%)142
**Infected/family infected**					
	Yes	17 (1.6%)	34 (3.8%)	1 (0.3%)	13 (4.4%)
	No	1,024 (98.4%)	857 (96.2%)	345 (99.7%)	283 (95.6%)
**Colleague infected**					
	Yes	235 (22.6%)	299 (33.6%)	59 (17.1%)	119 (40.2%)
	No	806 (77.4%)	592 (66.4%)	287 (82.9%)	177 (59.8%)
**Friend infected**					
	Yes	80 (7.7%)	113 (12.7%)	20 (5.8%)	35 (11.8%)
	No	961 (92.3%)	778 (87.3%)	326 (94.2%)	261 (88.2%)
**Neighborhood infected**					
	Yes	141 (13.5%)	184 (20.7%)	34 (9.8%)	58 (19.6%)
	No	900 (86.5%)	707 (79.3%)	312 (90.2%)	238 (80.4%)
**Age group (in years)**					
	18–30	450 (43.2%)	433 (48.6%)	142 (41.0%)	137 (46.3%)
	31–40	323 (31.0%)	294 (33.0%)	102 (29.5%)	89 (30.1%)
	41 and above	268 (25.7%)	164 (18.4%)	102 (29.5%)	70 (23.6%)
**Self-perceived current health status**					
	Not good	3 (0.3%)	53 (5.9%)	2 (0.6%)	15 (5.1%)
	Normal	300 (28.8%)	612 (68.7%)	82 (23.7%)	202 (68.2%)
	Good	738 (70.9%)	226 (25.4%)	262 (75.7%)	79 (26.7%)

### Model Development

**Figure 2:**
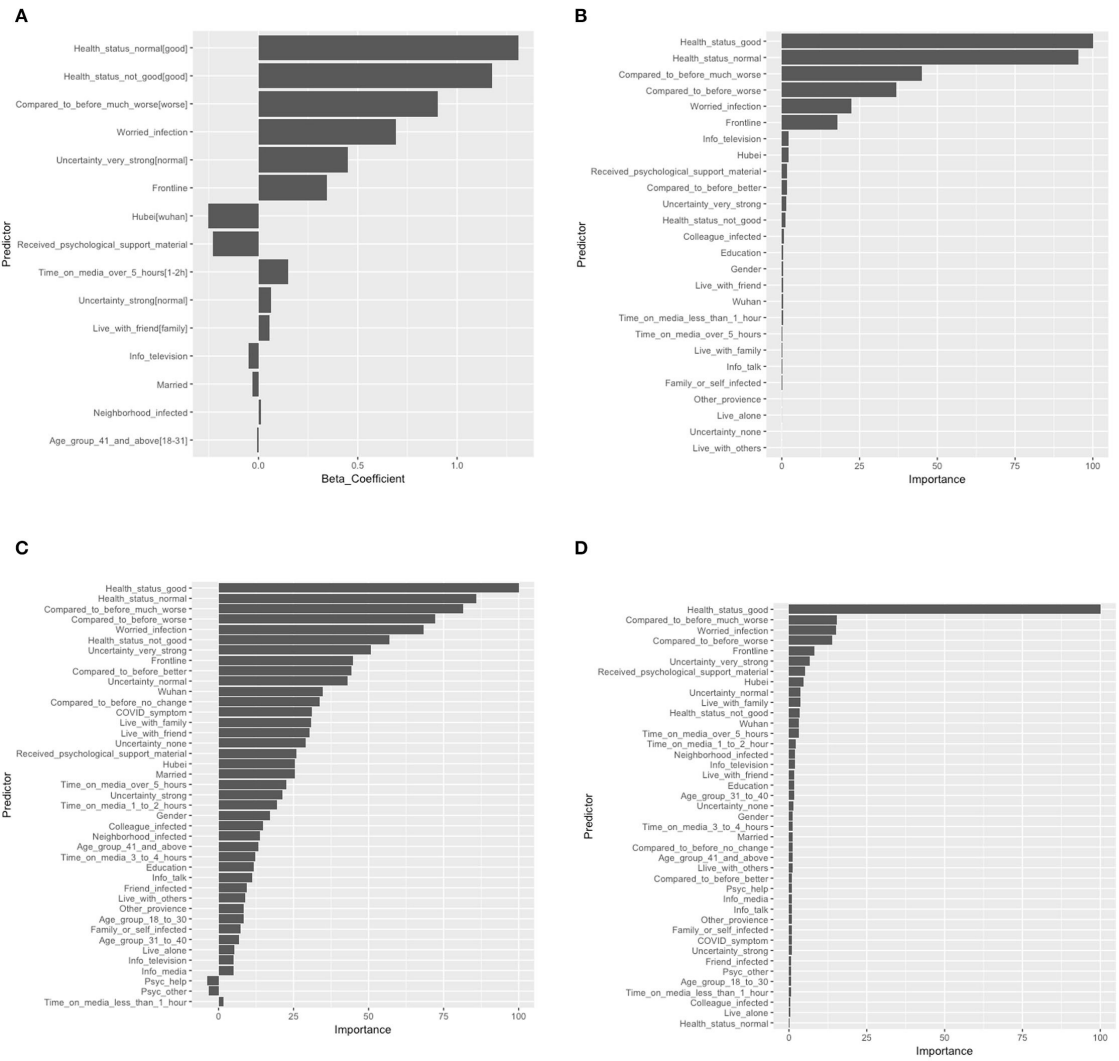
Feature weights and contributions to the models: **(A)** logistic regression with LASSO; **(B)** decision tree; **(C)** random forest; and **(D)** gradient-boosting tree. The beta coefficient in logistic regression with LASSO and the importance of variables scaled by the maximum value of 100 in the decision tree, random forest, and gradient-boosting tree were shown. LASSO, Absolute shrinkage and selection operator; [], reference variable.

The final logistic regression with LASSO selected 10 risk predictors and five protective predictors out of 36 potential predictors. The logistic model showed that self-perceived poor health [odds ratio (OR): 3.25 ref: self-perceived good health], self-perceived normal health (OR: 3.70 ref: self-perceived good health), self-perceived health status were much worse than before (OR: 2.47 ref: self-perceived health worse than before), worried about infection (OR: 2.00), very strong level of uncertainty regarding COVID-19 control (OR: 1.57 ref: normal level of uncertainty toward COVID-19 control), and working on the frontline (OR: 1.41) were the top five risk predictors. The model also identified two protective predictors: resided in Hubei province (OR: 0.78 ref: resided in Wuhan city) and received any form of psychological support material (OR: 0.80). Among all predictors identified by tree-based learning methods, self-perceived health status factors always occupied the top five most important predictors, followed by worried about infection, working on the frontline, a very strong level of uncertainty about control of the pandemic, receiving any form of psychological support material, and COVID-like symptoms ranked predictors' importance from tree-based methods generally matched the logistic with the LASSO model. These items also had high estimated ORs that were reflected by the LASSO model. Features and predictor contributions are presented in Figure 2.

### Model Performance and Evaluation

**Figure 3:**
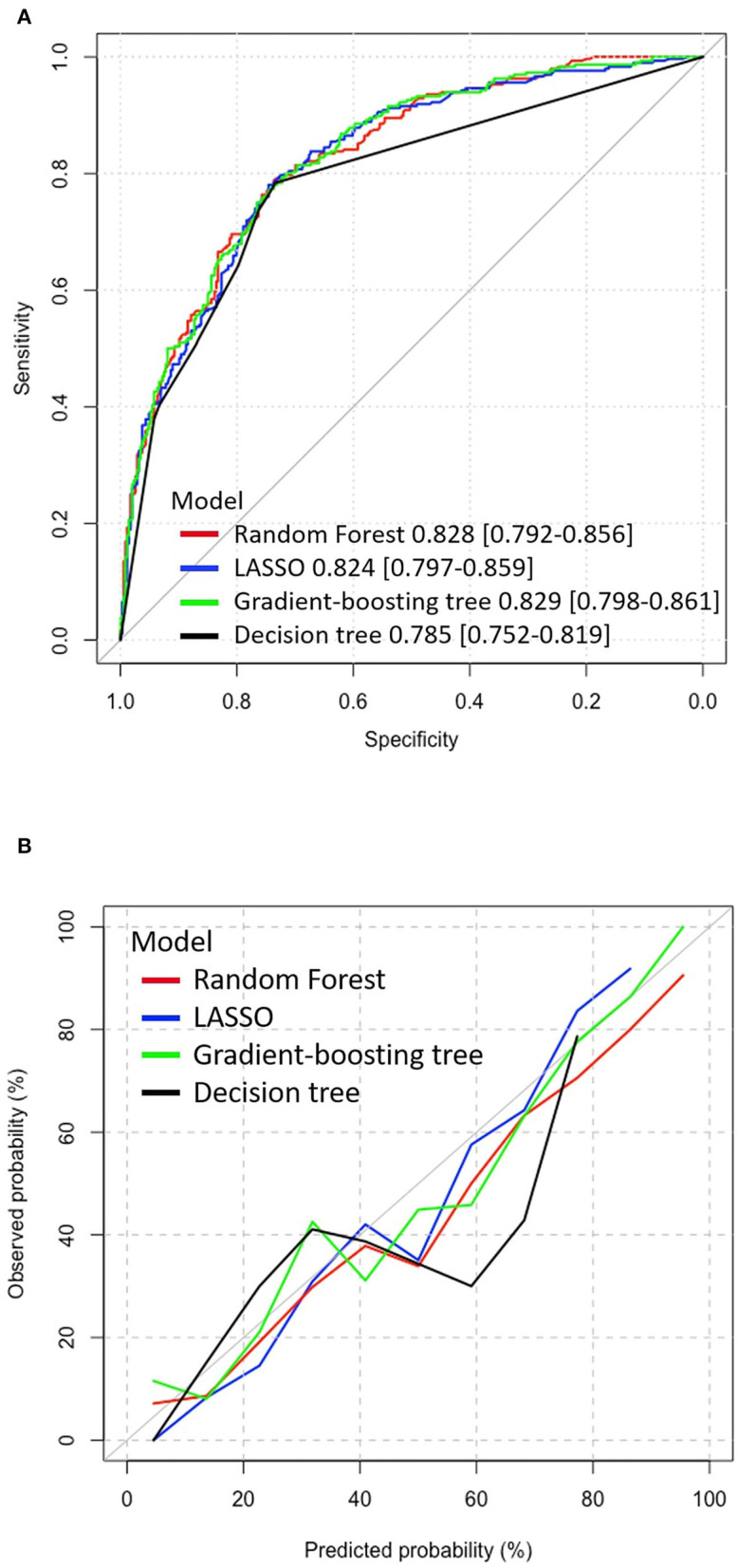
ROC curve and calibration plot of models in the test dataset. **(A)** The ROC curve of the models, *X*-axis: specificity, *Y*-axis: sensitivity. The AUC [95% CI] of the models; random forest: 0.828 [0.792–0.856], logistic regression with LASSO: 0.824 [0.797–0.859], gradient-boosting tree: 0.829 [0.798–0.861], and decision tree: 0.785 [0.752–0.819]. **(B)** Calibration plot, *X*-axis: probabilities estimated by machine learning models, *Y*-axis: observed probabilities of outcome. ROC, Receiver operating characteristic; AUC, Area under the curve; LASSO, Absolute shrinkage and selection operator.

As for prediction accuracy, the AUC [95% CI] of these machine learning models were as follows: logistic regression with LASSO, 0.824 [0.792–0.856]; random forest, 0.828 [0.797–0.859]; gradient-boosting tree, 0.829 [0.798–0.861]; and decision tree, 0.785 [0.752–0.819]. Based on the ROC analyses, there were significant differences in the AUC between the decision tree and the other three models (see Supplementary Table 2). The gradient-boosting tree showed higher overall accuracy with a slight advantage over the random forest and the LASSO model. The ROC curves for each model are shown in Figure 3A. To visualize the model fit, a calibration plot was carried out in Figure 3B. Overall all four models were underestimated after a predicted probability excess of 40%, considering all calibration lines were below the diagonal. The LASSO model, gradient-boosting tree, and random forest were calibrated very well although there were some fluctuations at predicted probability around 40–50%. The decision tree fitted poorly, which was overestimated and underestimated in predicting depression symptoms.

### Clinical Significance and Utility

**Figure 4:**
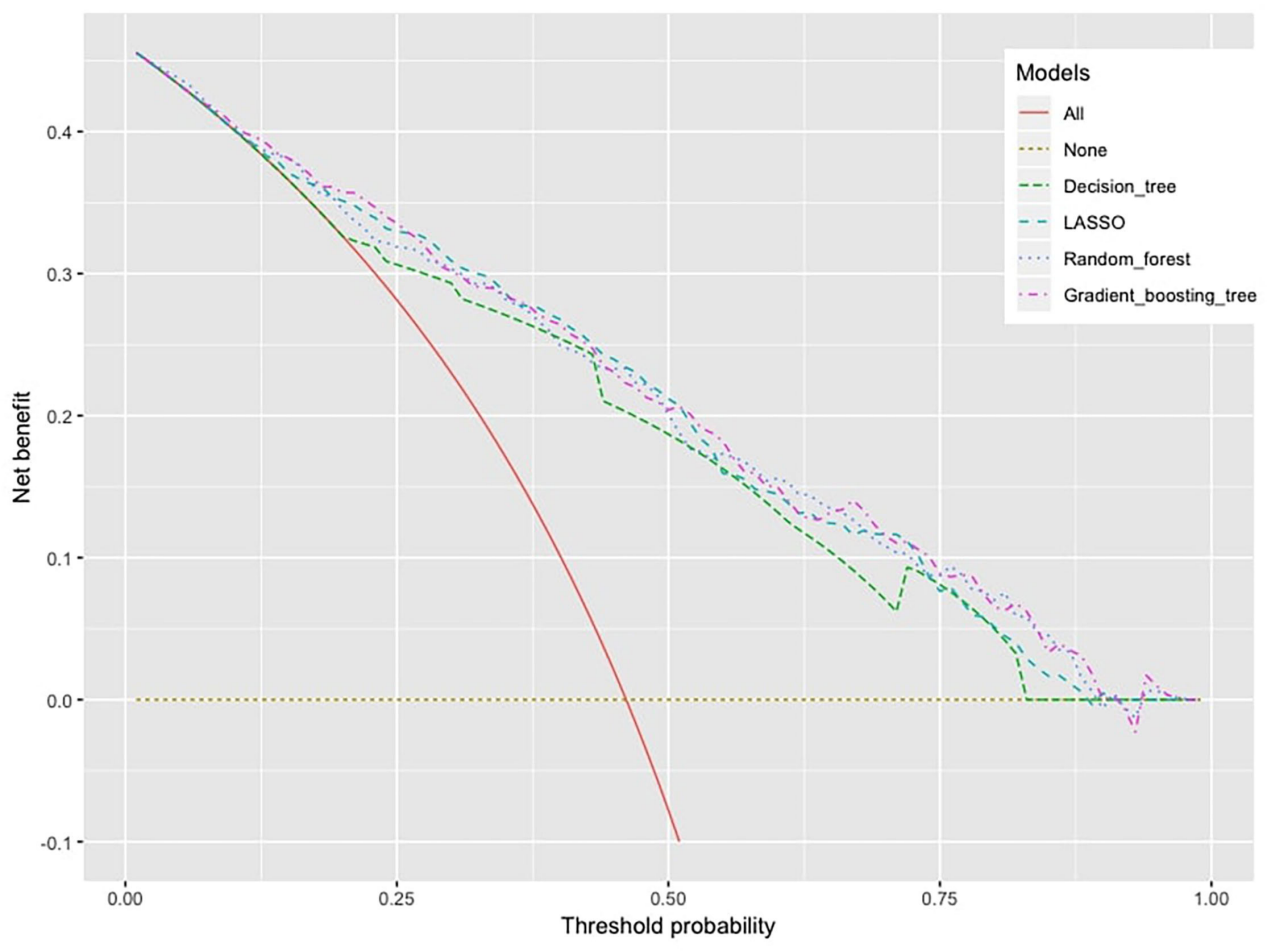
Decision curve analysis, *X*-axis: threshold probability for machine learning models to make a prediction, *Y*-axis: net benefit.

The decision curve analysis in Figure 4 showed that all models are clinically significant because the net-benefit values of the models were much higher than all-treatment and non-strategy. Again, the decision tree model had the lowest clinical value, which was expected due to its predictive performance. It is difficult to tell the difference in the net-benefit values among the rest of the three machine learning models, but it looks like ensemble tree-based learning methods (gradient-boosting tree and random forest) were slightly higher than the LASSO model.

## Discussion

This study successfully applied machine learning techniques to predict depression symptoms with reasonable accuracy and net benefit. In addition to the identified risk exposures that were already confirmed in previous studies [e.g., working on the frontline (11), worry about infection (12), and location of residence (11)], several hidden predictors associated with the mental health outcomes were uncovered which could be meaningful in constructing interventions. The decision curve analysis further suggested utility in mental screening implications.

As for the practice of “psychological first aid,” machine learning models identified several potential predictors that implied some possible mental interventions for healthcare workers. Information overload refers to the amount of news received that exceeds the limit of an individual's information processing capacity (44) and has been frequently studied in its association with the mental wellbeing of the general public during the COVID-19 pandemic (45–47). This study supported the existence of such an association between information overload and depression among healthcare workers and recognized several possible information overload thresholds. The LASSO model identified that an individual who spends over 5 h seeking COVID-19 information has higher odds of developing depression symptoms compared to those spending 1–2 h in obtaining COVID-19 information. Tree-based variable selection methods also identified spending 1–2 h obtaining COVID-19 information and spending over 5 h are paired predictors for depression symptoms. Moreover, all methods identified that receiving any form of psychological support material (both paper-based and media-based) can serve as important self-help therapy against depression. As several studies urged self-help strategies and social/mental health supports for healthcare workers during the pandemic (48–50), offering psychological support material can potentially be one feasible self-help solution. There are many advantages to offering psychological support material as a self-help intervention during the COVID-19 pandemic. Due to the high contagion of the virus and strict quarantine policy, traditional face-to-face psychotherapy is difficult to implement. Offering self-help psychological support material is an immediate intervention with minimum psychological therapist contact and increases the cost-effectiveness of the treatment. Plus, self-help interventions appeared to be the preferred option against depression over antidepressant medications (51). For healthcare workers who are worried about medication side effects or unwilling to show signs of mental hardship during employment, providing mental health/wellness pamphlets to all healthcare workers would be an appropriate resource for everyone.

### Strengths and Implications

This study has some major strengths compared with other COVID-related mental health studies. As far as we know, this is the first study to apply machine learning prediction models focusing on depression symptoms in healthcare workers during the COVID-19 outbreak. A sample size of 2,574 with 1,187 events allows for multifold cross-validation to prevent model overfitting and uses a separate test dataset to evaluate predictive performance.

Tree-based machine learning methods have advantages of modeling variable to variable interactions (52, 53), complex data (54), and nonparametric data (55). For example, complex categorical variables (in this study: self-perceived health factors, media factors, and psychological services factors) were usually ignored or excluded from previous COVID-19 mental health survey studies (11–13). This is because conventional statistical approaches (such as univariate or multivariate logistic regression) that were commonly adopted either are impossible to model hundreds of interactions among variables or have to follow strict data distribution assumptions. Hence, tree-based machine learning models not only provide more accurate predictions but also provide a different angle by looking at survey data using a data-driven approach instead of a traditional hypothesis-driven approach.

As for the screening implication, the researchers believe this machine learning-based prediction model would play a crucial role as an efficient early screening tool and report information to hospitals about healthcare workers' mental status. Especially when background knowledge of depression caused by the outbreak is lacking, machine learning models could make predictions by using easily acquired information such as demographic data, work-related factors, outbreak factors, or self-perceived factors. They may enable hospitals to quickly collect depression statistics and accurately identify individual at-risk workers for targeted interventions and proper management. The other advantage is that giving actual probabilities of depression symptoms is more informative to healthcare workers than Yes/No answers. Healthcare workers can self-evaluate their current mental status through the depression probabilities and then decide whether they need professional mental health support. These machine learning techniques can be easily implemented in software such as the WeChat mini program and Weibo. Further to enhance the use, allowing some programs to extract healthcare workers' basic demographic data would be necessary. Although there are several well-established depression screening tools (24, 56, 57), none of them is designed for use during a pandemic situation. Taking a step back, even if machine learning models do not show superior performance over conventional screening tools, combined use with conventional tools could still be very beneficial because they may provide more diagnostic information specifically in a public health emergency setting.

### Limitations

This study has several major limitations which could point the direction for future research. First, large sample size and ethnic diversity of participants are always required for cross-site validating of model performance. It is often difficult to obtain a large sample at one geographic location, and even more difficult to contain participants from ethnic minorities or other races globally. In our survey, 1,102 (42.81%) healthcare workers were from Wuhan city with predominantly Han Chinese. To address this problem, integrating data from international sites would be essential for future work to conduct cross-site model validations. Machine learning models can be trained at one or several independent sites in one country and tested at different sites abroad. The advantage of such cross-site validation is it can correct overfitting problems arising at a single geographic location. Cross-site validation technique had been successfully applied to the classification of mental disorders such as schizophrenia classification using MRI data and showed promising performance. Rozycki et al. (58) used data from 941 participants from 5 sites (location: China, United States, and Germany) to build a linear support vector machine that discovered important neuroanatomical biomarkers of patients with schizophrenia and find robust generalizability of these biomarkers across different sites. Zeng et al. (59) cross-validated deep learning models from 7 sites located in both China and the United States; and found reliable connectome patterns of schizophrenia across independent sites. The above studies did both pooling classification and leave-site-out validation and obtained high classification accuracy (AUC around 0.8). These cross-site validation methods may transfer to the field of depression disorders to construct predictive models and increase the generalizability of the predictive model across the world.

The study is also lacking longitudinal follow-up because the epidemic in China from the outbreak to the control happened quickly. As the global epidemic is prolonged, depression predictions that focus on the longitudinal progression patterns among healthcare workers are worth exploring. Hence, more longitudinal survey “waves” should be carried out to capture time-series information on potential risk predictors. Su et al. (60) did 5 waves of the same survey that aimed to develop machine learning predictive models on depression symptoms among elderly people. The survey contains the exact same categories of questions such as demographics and health-related risk factors. The long short-term memory model was used to predict the values of predictors in the next 2 years, then 6 machine learning models were applied to make depression symptoms predictions. The novelty of longitudinal survey study is it allows machine learning models to merge and characterize the complex interaction between time patterns and predictors. Such successful capture of correlation between static data (predictors) and dynamic data (time) can extend the prediction scope from real-time outcome prediction to outcome's future tendency prediction. If the same longitudinal survey could be done for healthcare workers, it will give researchers opportunities to learn about future depression tendencies influenced by COVID-19 and the progression mechanism between important predictors and depression symptoms in the flow of time.

Last but not the least, future application of machine learning models in predicting depression symptoms in general diagnostic settings remains unclear. Doctors may still prefer making diagnostic decisions based on more traditional criteria. The “black box” nature of machine learning algorithms is sometimes difficult to interpret irrelevant psychological factors. It should also be recognized that the rule played by the machine learning-based predictions model is the decision support system. Machine learning-based predictions model can capture valuable predictors out of high dimensional information provided to psychiatrists and doctors at the outbreak of public health emergency.

## Conclusion

This study shows that machine learning prediction models are suitable for making mentally at-risk healthcare worker predictions in a public health emergency setting. As the COVID-19 pandemic change the way people live and work: minimal contact, strict working condition, and growing media influences; the “psychological first aid” can be focused on preparing immediate noncontact psychological consulting material (both paper-based and media-based); and controlling media consumption time avoiding information overload. The application of machine learning models could support hospitals' and healthcare workers' decision-making on early psychological interventions and proper mental health management. Further study of machine learning models predicting high-risk depression symptoms among healthcare workers in cross-site validation is warranted.

## Data Availability Statement

The raw data supporting the conclusions of this article will be made available by the authors, without undue reservation.

## Ethics Statement

All study participants provided informed consent, and the study design was approved by the Institutional Review Board at Renmin Hospital of Wuhan University (No. WDRY2020-K004). The patients/participants provided their written informed consent to participate in this study.

## Author Contributions

BXY and ZL have full access to all the data in the study, take data analysis, and supervision. ZZ and DL: conceptualization, methodology, and writing-original draft. ZZ: software and validation. DL, BXY, and ZL: writing-reviewing and editing. All authors approved the submitted version.

## Funding

This study was supported by the grant: National Key R&D Program of China (2018YFC1314600).

## Conflict of Interest

The authors declare that the research was conducted in the absence of any commercial or financial relationships that could be construed as a potential conflict of interest.

## Publisher's Note

All claims expressed in this article are solely those of the authors and do not necessarily represent those of their affiliated organizations, or those of the publisher, the editors and the reviewers. Any product that may be evaluated in this article, or claim that may be made by its manufacturer, is not guaranteed or endorsed by the publisher.

## Abbreviations

COVID-19: 2019 novel coronavirus
PHQ-9: 9-item patient health questionnaire
LASSO: Least absolute shrinkage and selection operator
ROC: Receiver operating characteristic
AUC: Area under the curve.
