# Supplementary material for: Machine Learning-Based Prediction Models for Depression Symptoms Among Chinese Healthcare Workers During the Early COVID-19 Outbreak in 2020: A Cross-Sectional Study
Source: Front Psychiatry. 2022 Apr 29;13:876995. doi: 10.3389/fpsyt.2022.876995 (PMC9106105; doi:10.3389/fpsyt.2022.876995)
Supplement: Supplementary file 1 [file Data_Sheet_1.docx]

**Supplementary Material**

**Supplementary Table 1.** Hyperparameters in machine learning models

| **Model** | **Hyperparameter** | **Value** |
| --- | --- | --- |
| Lasso | Alpha | 1 |
|  | Minimal lambda | 0.0076 |
| Random forest | Number of variables randomly sampled as candidates at each split (mtry) | 2 |
|  | Number of trees | 1000 |
| Gradient boosting tree | Minimum number of observations in the terminal nodes (n.minobsinnode) | 5 |
|  | Number of trees | 1000 |
|  | Shrinkage | 0.01 |
|  | Max tree depth | 2 |
| Decision tree | Complexity parameter (cp): minimum improvement of a split by a factor of cp is not attempted | 0.00786 |

**Supplementary Table 2.** C-statistics for each model

| **Model** | **C-statistics [95%CI]** | ***P*-value** |
| --- | --- | --- |
| Lasso | 0.824 [0.792-0.856] | 8.749e-6 |
| Random forest | 0.828 [0.797-0.859] | 2.283e-6 |
| Gradient boosting tree | 0.829 [0.798-0.861] | 9.362e-7 |
| Decision Tree | 0.785 [0.752-0.819] | Reference |

The C-statistics were compared to decision tree by Delong test.
